# The DNA loop release factor WAPL suppresses Epstein-Barr virus latent membrane protein expression to maintain the highly restricted latency I program

**DOI:** 10.1371/journal.ppat.1012525

**Published:** 2024-09-06

**Authors:** Laura A. Murray-Nerger, Davide Maestri, Xiang Liu, Zhixuan Li, Nina R. Auld, Italo Tempera, Mingxiang Teng, Benjamin E. Gewurz

**Affiliations:** 1 Division of Infectious Diseases, Department of Medicine, Brigham and Women’s Hospital, Boston, Massachusetts, United States of America; 2 Department of Microbiology, Harvard Medical School, Boston, Massachusetts, United States of America; 3 Harvard Program in Virology, Boston, Massachusetts, United States of America; 4 Broad Institute of Harvard and MIT, Cambridge, Massachusetts, United States of America; 5 The Wistar Institute, Philadelphia, Pennsylvania, United States of America; 6 Department of Pharmacy and Biotechnology, University of Bologna, Bologna, Italy; 7 Department of Biostatistics and Bioinformatics, H. Lee Moffitt Cancer Center and Research Institute, Tampa, Florida, United States of America; University of Wisconsin-Madison, UNITED STATES OF AMERICA

## Abstract

Epstein-Barr virus (EBV) uses latency programs to colonize the memory B-cell reservoir, and each program is associated with human malignancies. However, knowledge remains incomplete of epigenetic mechanisms that maintain the highly restricted latency I program, present in memory and Burkitt lymphoma cells, in which EBNA1 is the only EBV-encoded protein expressed. Given increasing appreciation that higher order chromatin architecture is an important determinant of viral and host gene expression, we investigated roles of Wings Apart-Like Protein Homolog (WAPL), a host factor that unloads cohesin to control DNA loop size and that was discovered as an EBNA2-associated protein. WAPL knockout (KO) in Burkitt cells de-repressed LMP1 and LMP2A expression, but not other EBV oncogenes, to yield a viral program reminiscent of EBV latency II, which is rarely observed in B-cells. WAPL KO also increased LMP1/2A levels in latency III lymphoblastoid cells. WAPL KO altered EBV genome architecture, triggering formation of DNA loops between the LMP promoter region and the EBV origins of lytic replication (*oriLyt*). Hi-C analysis further demonstrated that WAPL KO reprogrammed EBV genomic DNA looping. LMP1 and LMP2A de-repression correlated with decreased histone repressive marks at their promoters. We propose that EBV coopts WAPL to negatively regulate latent membrane protein expression to maintain Burkitt latency I.

## Author summary

EBV is a highly prevalent herpesvirus etiologically linked to multiple lymphomas, gastric and nasopharyngeal carcinomas, and multiple sclerosis. EBV persists in the human host in B-cells that express a series of latency programs, each of which is observed in a distinct type of human lymphoma. The most restricted form of EBV latency, called latency I, is observed in memory cells and in most Burkitt lymphomas. In this state, EBNA1 is the only EBV-encoded protein expressed to facilitate infected cell immunoevasion. However, epigenetic mechanisms that repress expression of the other eight EBV-encoded latency proteins remain to be fully elucidated. We hypothesized that the host factor WAPL might have a role in restriction of EBV genes, as it is a major regulator of long-range DNA interactions by negatively regulating cohesin proteins that stabilize DNA loops, and WAPL was found in a yeast 2-hybrid screen for EBNA2-interacting host factors. Using CRISPR together with Hi-ChIP and Hi-C DNA architecture analyses, we uncovered WAPL roles in suppressing expression of LMP1 and LMP2A, which mimic signaling by CD40 and B-cell immunoglobulin receptors, respectively. These proteins are expressed together with EBNA1 in the latency II program. We demonstrate that WAPL KO changes EBV genomic architecture, including allowing the formation of DNA loops between the *oriLyt* enhancers and the LMP promoter regions. Collectively, our study suggests that WAPL reinforces Burkitt latency I by preventing the formation of DNA loops that may instead support the latency II program.

## Introduction

Epstein-Barr virus (EBV) infects >95% of adults and causes ~200,000 cancers/year, including Burkitt and Hodgkin lymphomas and nasopharyngeal and gastric carcinomas [1–5]. The double-stranded DNA EBV genome is circularized and chromatinized upon infection. EBV uses the pre-latency IIb and latency III programs in newly infected B-cells [6–8], the latter of which expresses six Epstein-Barr nuclear antigens (EBNA) and two latent membrane proteins (LMP), LMP1 and LMP2A, which mimic signaling by CD40 and immunoglobulin receptors, respectively [9–11].

**Fig 1:**
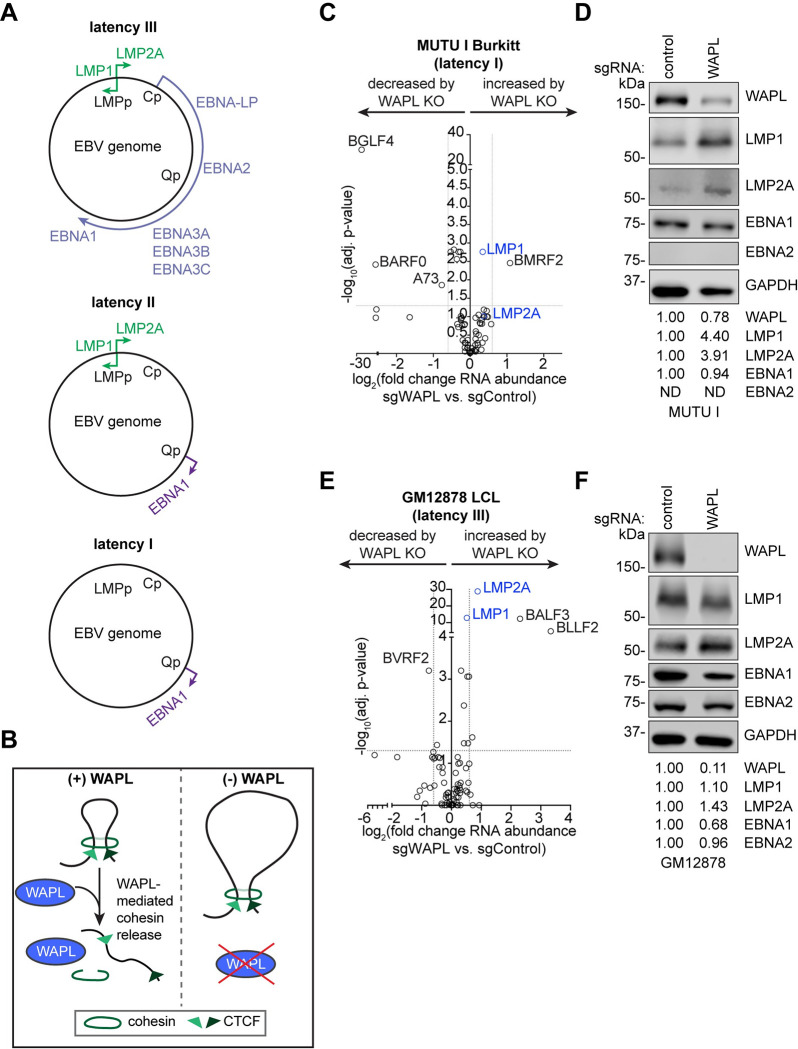
WAPL negatively regulates LMP1 and LMP2A expression. **(A)** Schematic diagram of EBV latency programs. **(B)** Schematic of WAPL antagonism of cohesin-mediated DNA loop formation. WAPL releases cohesin to promote dissolution of chromatin loops. Upon WAPL KO, cohesin occupancy on chromatin increases, resulting in larger DNA loops. **(C, E)** Volcano plots of RNA-seq analysis visualizing -log_10_(adj. p-value) vs. log_2_(fold change of EBV mRNA abundance) from (C) Cas9+ MUTU I Burkitt lymphoma cells and (E) Cas9+ GM12878 LCLs expressing WAPL vs. control sgRNAs, from n = 3 independent biological replicates. **(D, F)** Immunoblot analysis of whole cell lysates (WCL) from (D) MUTU I cells and (F) GM12878 LCLs expressing control or WAPL sgRNAs, as indicated, representative of n = 3 biological replicates. Shown below are densitometry values that were normalized to GAPDH loading control, with control levels normalized to 1. ND indicates not detected.

Microenvironmental cues trigger EBV switching to latency IIa, where the Q promoter (*Qp*) and LMP promoters (*LMPp*) drive expression of EBNA1 and LMP1/2A, respectively. Cytokines IL-15 and IL-21 downmodulate EBNA expression while supporting LMP1 expression [12–15]. Latency IIa B-cells differentiate into memory cells, the EBV reservoir, where, in latency I, only EBNA1 is expressed [1]. Hodgkin Reed-Sternberg cells use latency IIa [1,2,16], whereas Burkitt lymphoma and gastric carcinoma use latency I [17] (**Fig 1A**). Much remains to be learned about the latency IIa to I transition and chromatin-based mechanisms that maintain latency I.

Three-dimensional genome architecture is a major determinant of EBV gene expression [18–21]. The cohesin complex (SMC1, SMC3, and RAD21) forms a ring-shaped structure that encircles DNA to mediate long-range genomic interactions [22]. CTCF and cohesin are loaded onto discrete EBV and host genomic sites [18,21,23–31]. DNA loops juxtapose the EBV genomic origin of plasmid replication (*OriP)* enhancer with *Cp* and the *LMPp* region to support latency III [23,30,32]. However, the *OriP/LMPp* loop is observed in latency I, where it is not sufficient to drive LMP1/2A expression [30].

Several factors limit DNA loop size [21,23–25]. First, paired CTCF sites block cohesin-driven loop extrusion to anchor DNA loops. Second, WAPL (wings apart-like protein homolog) limits DNA loop size [33–36]. Consequently, large DNA loops are observed in WAPL deficient cells [34] (**Fig 1B**). While WAPL was discovered in a yeast-2 hybrid screen for EBNA2 associated factors [37], WAPL roles in EBV-infected cells are unstudied.

Here, we tested the hypothesis that EBV utilizes WAPL to regulate viral gene expression. Burkitt WAPL knockout (KO) de-repressed LMP1/2A, but not other EBV latency genes, suggestive of a switch towards latency IIa. WAPL KO altered specific EBV genomic DNA loops, especially at the *LMPp* and *oriLyt* enhancers.

## Results

### WAPL is necessary for maintenance of EBV latency I

To test the role of WAPL in EBV gene regulation, we knocked out WAPL in latency I Burkitt MUTU I or latency III GM12878 lymphoblastoid (LCL) cells (**S1A–S1F Fig**). WAPL KO did not significantly alter MUTU I or GM12878 proliferation, even though it dramatically altered nuclear morphology (**S1A–S1F Fig**), consistent with prior studies in EBV-negative cancer cells [33,34].

To define how WAPL KO affects human and EBV gene expression, we performed RNA sequencing (RNA-seq) in WAPL KO and control MUTU I and GM12878. While most EBV gene levels were unchanged by WAPL KO, MUTU I LMP1 and LMP2A levels increased (**Figs 1C and S1I and S1 Table**). By contrast, EBNA2 levels did not substantially increase, suggesting an alternative, latency IIa-like mechanism of LMP1/2A induction (**Figs 1C–1D, S1H, and S1I**). WAPL KO did not significantly increase expression of most EBV lytic genes or EBV genome copy number (**Figs 1C–1D and S1G-S1I and S1 Table**). WAPL KO modestly impacted GM12878 LMP1/2A abundances but did not significantly alter EBNA2 or EBNA1 (**Figs 1E–1F and S1I and S1 Table**). WAPL mRNA and protein levels were approximately 50% lower in GM12878 than in MUTU I (**S2A–S2E Fig**), which correlated with a comparatively modest effect of WAPL depletion on GM12878 LMP1/2A abundances.

We interrogated WAPL KO effects on host gene expression. LMP1/NF-κB targets [38] were amongst the most highly induced by WAPL KO in MUTU I, including the chemokines *CCL3*, *CCL4*, and *CCL22* and the anti-apoptotic cIAP2 (*BIRC3*) and BFL1 (*BCL2A1*) (**S3A Fig**). Gene ontology analyses identified that chemotaxis/chemokine pathways were most highly upregulated by Burkitt WAPL KO (**S3B Fig**). GM12878 WAPL KO also upregulated *CCL3* and *CCL4*, together with antiviral type II interferon responses (**S3C and S3D Fig**).

### Subcellular distribution of de-repressed LMP1 and LMP2A

**Fig 2:**
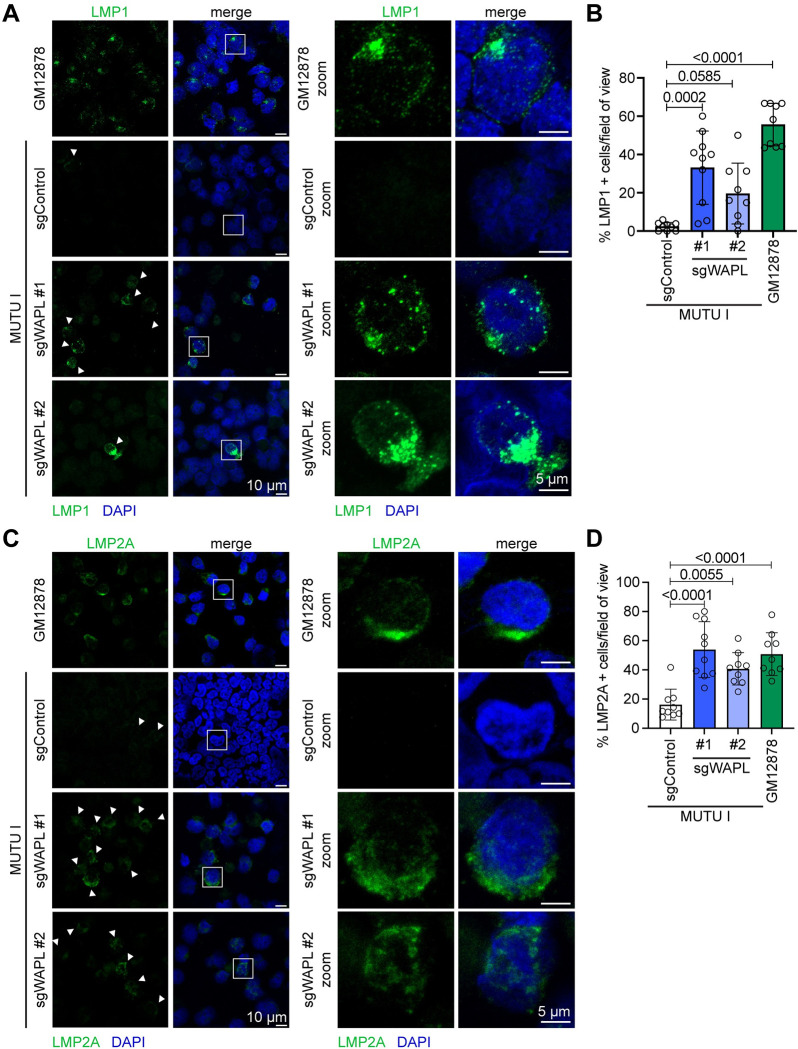
Subcellular distribution of LMP1 and LMP2A de-repressed by WAPL KO. **(A)** Representative immunofluorescence images from n = 3 biological replicates of anti-LMP1 (green) vs. nuclear DAPI (blue) staining of Cas9+ MUTU I cells that expressed control or WAPL sgRNAs, as indicated. Shown at right are zoomed images of a representative cell (indicated by the white box). **(B)** Mean ± standard deviation (SD) percentage of LMP1+ cells per field of view, from n = 3 fields of view from each of three biological replicates. *P*-values shown as calculated by one-way ANOVA. **(C)** Representative immunofluorescence images from n = 3 biological replicates of anti-LMP2A (green) vs. nuclear DAPI (blue) staining of Cas9+ MUTU I cells that expressed control or WAPL sgRNAs with zoomed images presented to the right, as in (A). **(D)** Mean ± SD percentage of LMP2A+ cells per field of view, from n = 3 fields of view from each of three biological replicates. *P*-values shown as calculated by one-way ANOVA.

LMP1/2A signal from plasma membrane and endosomal sites where they form puncta/membrane caps [39–44]. LMP1 puncta were observed in a significant proportion of WAPL KO, but rarely in control, MUTU I (**Fig 2A and 2B**). Similar results were obtained for LMP2A (**Fig 2C and 2D**).

Since B-cell latency IIa models are unavailable, we asked whether LMP1/2A formed membrane puncta in WAPL KO P3HR-1 Burkitt cells, which harbor an EBNA2 deletion [45–48]. WAPL KO de-repressed LMP1/2A in P3HR-1 and formed characteristic puncta (**S4A–S4E Fig**), indicating that WAPL is required to repress Burkitt LMP expression even without EBNA2. However, the percentage of cells that de-repressed LMP1/2A was lower than in MUTU I. This may be related to disruption of EBV genomic architecture by the P3HR-1 deletion.

### WAPL regulates LMP region looping

**Fig 3:**
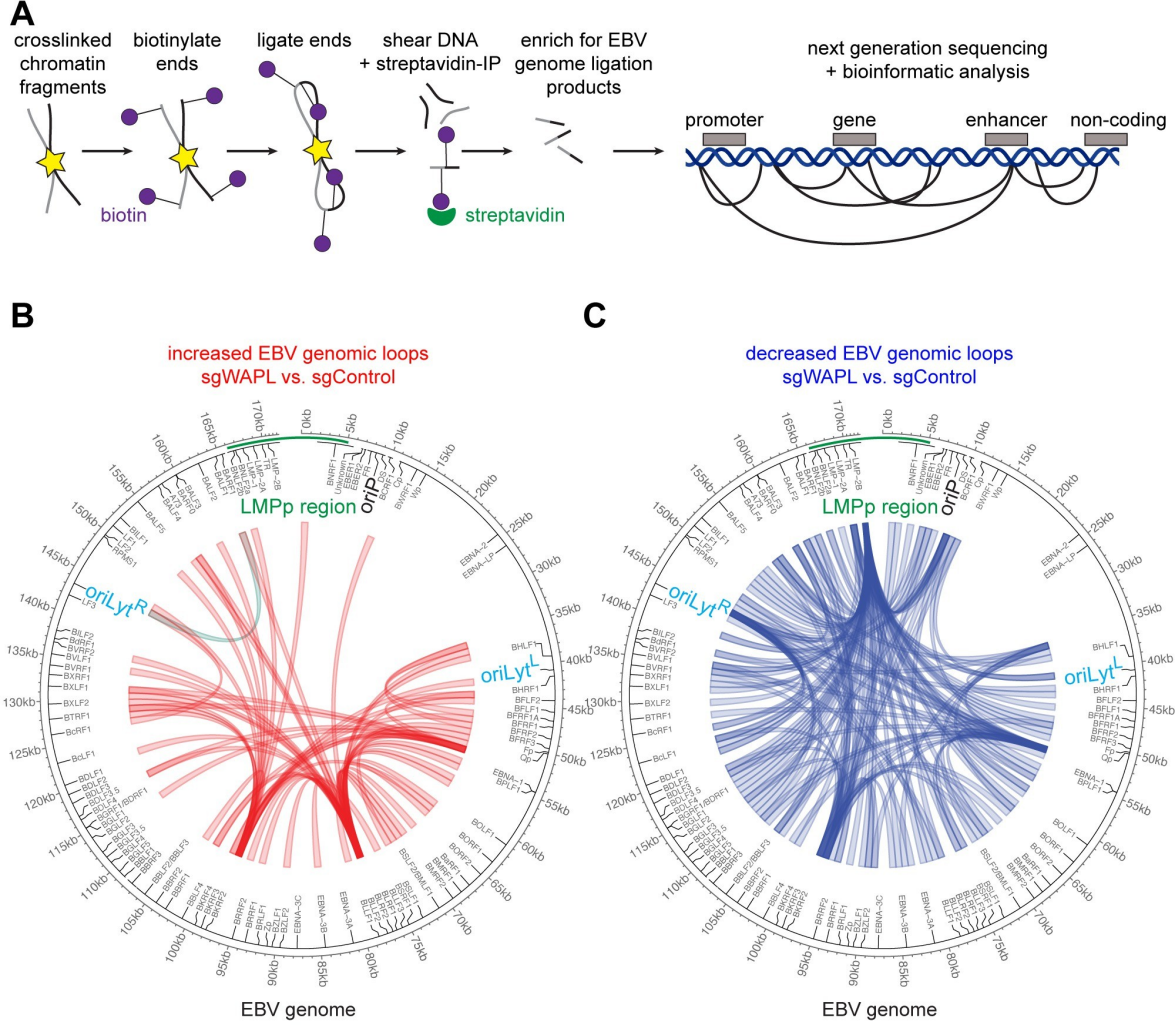
WAPL KO alters higher order latency I Burkitt EBV genome conformation. **(A)** Schematic of Hi-C workflow and output. Exposed DNA ends were biotinylated and then ligated to capture close DNA contacts. Ligated DNA was sheared, and biotinylated DNA was captured via streptavidin. EBV DNA was captured to enhance viral DNA Hi-C signal. **(B)** Hi-C maps of EBV genomic loops that were enriched in Cas9+ MUTU I cells expressing WAPL vs. control sgRNAs, from n = 2 biological replicates. LMPp and oriLyt regions are indicated. **(C)** Hi-C maps of EBV genomic loops that were depleted in Cas9+ MUTU I cells expressing WAPL vs. control sgRNAs, from n = 2 biological replicates, as in (B).

To test the hypothesis that WAPL KO altered EBV genomic architecture to de-repress LMP1/2A, we performed EBV genomic Hi-C, which measures long-range DNA contacts [27,49,50] (**Fig 3A**). At a FDR < 0.05 and Z-score > 1 cutoff, Hi-C identified 60 EBV genomic loops gained upon WAPL KO (**Fig 3B, S2 Table**), including between the LMP region and the rightward *oriLyt* (*oriLyt*^*R*^) enhancer. A loop was also gained between the LMP region and *BKRF2*, which looped to the *BLRF2* and *EBNA1* region (**Fig 3B**). WAPL depletion significantly decreased 138 EBV DNA loops (**Fig 3C, S2 Table**), including from the LMP region to multiple EBV genomic locations (**Fig 3C**).

**Fig 4:**
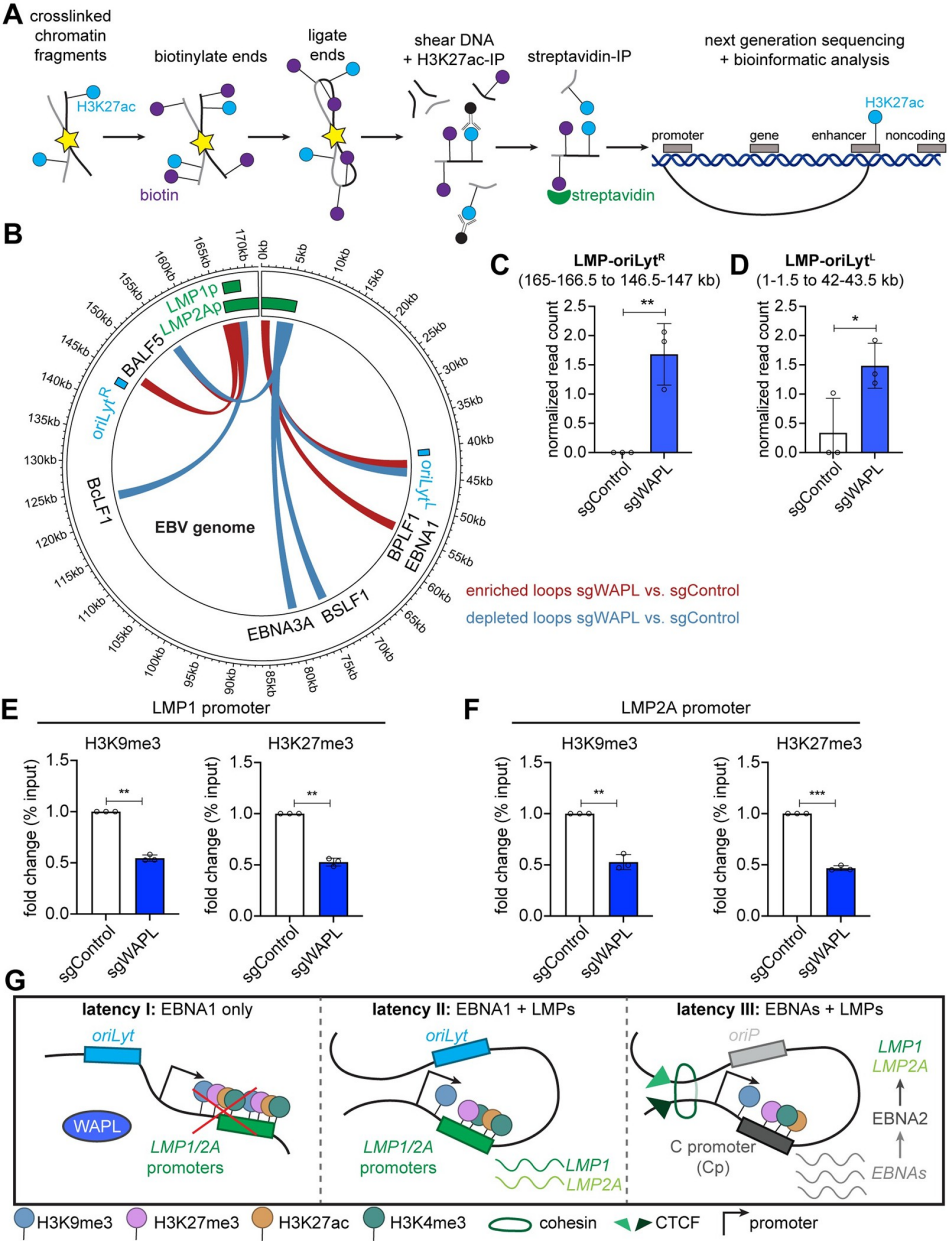
WAPL KO alters latency I Burkitt EBV genomic activated chromatin loops and represses LMP promoter epigenetic marks. **(A)** Schematic of H3K27ac HiChIP sample preparation and output. Chromatin was formaldehyde crosslinked and fragmented. Exposed DNA ends were biotinylated and then ligated to capture close DNA contacts. Ligated DNA was sheared, DNA was immunopurified by α-H3K27ac antibody, and biotinylated DNA was captured via streptavidin. **(B)** EBV genomic H3K27ac HiChIP map depicting loops enriched (red) or depleted (blue) in Cas9+ MUTU I cells expressing WAPL vs. control sgRNAs, from n = 3 biological replicates. **(C-D)** Normalized (C) LMP region-oriLyt^R^ loop and (D) LMP region-oriLyt^L^ loop read counts from n = 3 replicates, as in (B). EBV genome kilobase coordinates for each looping region are indicated at top. * *P* ≤ 0.05, ** *P* ≤ 0.01, as calculated by a two-tailed Student’s t-test. **(E-F)** ChIP-qPCR analysis of H3K9me3 and H3K27me3 abundances at the (E) LMP1 promoter and (F) LMP2A promoter in Cas9+ MUTU I cells expressing control or WAPL sgRNAs. Shown are the mean fold change of ChIP-qPCR values relative to input values ± SD from n = 3 biological replicates. Values from sgControl expressing cells were normalized to 1. ** *P* ≤ 0.01, *** *P* ≤ 0.001, as calculated by a two-tailed Welch’s t-test. **(G)** Model of WAPL effects on EBV genomic architecture. When present, WAPL releases cohesin (latency I), which enables the accumulation of repressive histone marks and inhibits LMP expression. In the absence of WAPL antagonism, a loop forms between the LMP promoter region and *oriLyt* regions. Juxtaposition of the *oriLyt* enhancer reduces repressive H3K9me3 and H3K27me3 marks and supports *LMP1* and *LMP2A* co-expression in the absence of EBNA2 (latency II). For reference, in latency III, an alternative loop forms between the *oriP* and the *Cp* to drive expression of all the EBNA genes.

We used HiChIP [51] to define how WAPL KO altered long-range EBV genomic interactions between areas of activated chromatin [52,53], marked by histone 3 lysine 27 acetyl (H3K27ac) (**Fig 4A**). HiChIP identified a higher frequency of interactions between LMP and both *oriLyt* regions (**Figs 4B–4D and S5A and S5B and S3 Table**). By contrast, WAPL KO decreased interactions between H3K27ac-marked LMP and other EBV genomic regions (**S5A and S5B Fig**). Both Hi-C and HiChIP detected an *oriLyt*^*R*^/LMP region loop upon WAPL KO. ChIP-qPCR highlighted that *LMPp* SMC1 and CTCF occupancy were significantly diminished by WAPL KO in MUTU I (**S6A Fig**).

We next characterized WAPL KO effects on *LMPp* region histone marks. WAPL KO significantly decreased repressive histone 3 lysine 9 and lysine 27 trimethylation (H3K9me3/H3K27me3) at both the *LMP1p* and *LMP2Ap* (**Fig 4E and 4F**). While polycomb repressive complex I mediated histone 2A lysine 119 monoubiquitination (H2AK119ub) represses Burkitt LMP1/2A [54], its *LMP1p* or *LMP2p* region levels were not significantly changed by WAPL KO (**S6B and S6C Fig**). WAPL KO did not significantly change activating H3K27ac marks at the *LMP1p* and decreased them at the *LMP2Ap*, while histone 3 lysine 4 trimethylation (H3K4me3) marks were not significantly altered at either site (**S6B and S6C Fig**). These results are consistent with a model where WAPL supports EBV latency I by altering EBV genomic structure to increase repressive *LMPp* H3K9me3 and H3K27me3 marks to maintain latency I (**Fig 4G**).

## Discussion

Much remains to be learned about epigenetic mechanisms that maintain latency I. We found the cohesin release factor WAPL represses LMP1/2A in Burkitt latency I and alters higher order EBV genomic architecture. WAPL KO triggered DNA loops between *oriLyt* and *LMPp*, decreased *LMPp* repressive H3K9me3/H3K27me3 marks, and de-repressed LMP1/2A co-expression, even without EBNA2. These results highlight an important WAPL role in preventing reversion to latency II.

WAPL loss permits cohesin to slide beyond human CTCF anchors and enlarges DNA loops [33]. Our findings suggest that WAPL likewise regulates EBV genome architecture. EBV genomic structure may be distinct between latency IIa germinal center B-cells and latency I memory B-cells. Future work will determine whether WAPL abundance or activity differs between these states. Since germinal center cytokine IL-21 boosts LMP1 expression in latency I [12], it may alter WAPL activity, potentially in a STAT3-dependent manner.

WAPL KO reduced *LMPp* histone repressive marks in latency I, suggesting that WAPL supports an EBV genomic configuration that represses LMP1/2A (**Fig 4G**). While WAPL KO may alter a host factor that itself alters *LMPp* epigenetic marks, WAPL KO did not alter expression of H3K9me3/H3K27me3 writers or erasers. Instead, WAPL may prevent *oriLyt/LMPp* loop formation. DNA loops between *oriLyt* and *LMPp* regions were described in gastric carcinoma and natural killer cells [25,55], but not previously in B-cells. Instead, in latency III, cohesin and CTCF bind to the LMP1/2A region to drive *OriP* enhancer and *LMPp* looping. However, the *OriP*/*LMPp* loop is present in MUTU I, where LMP1/2A are silenced [30], suggesting additional mechanisms repress LMP in latency I. Consistent with our finding that WAPL KO diminished *LMPp* cohesin occupancy, WAPL creates a pool of free cohesin. WAPL knockdown can alter cohesin occupancy and enhancer-promoter looping at human genomic sites [32]. We speculate that a similar mechanism accounts for the decrease in *LMPp* cohesin level. Furthermore, LCL SMC1 or RAD21 knockdown increases LMP1/2A levels [32], consistent with a model in which WAPL supports *LMPp* cohesin occupancy in latency I. Additional regulators, such as cytokines [12], likely work with DNA looping to regulate latency gene expression.

WAPL was discovered as an EBNA2 binding partner [37]. Since EBNA2 is a major inducer of LMP1/2A in EBV latency III, an intriguing possibility is that EBNA2 not only activates *LMPp* chromatin but also dismisses WAPL from *LMPp*. EBNA2 may alter EBV genomic architecture to reduce H3K9me3/H3K27me3 repressive marks in support of LMP expression in newly infected cells. In latency III, this mechanism may function with EBNA2-driven TET2 DNA hypomethylation [56,57]. Future work will determine whether EBNA2 alters EBV genomic looping. Our studies highlight a correlation between WAPL depletion and altered EBV genomic looping, and further studies are needed to define a direct WAPL role in control of EBV genomic architecture.

In conclusion, our data suggest that EBV coopts WAPL in latency I to regulate higher order EBV genome architecture to restrict LMP1/2A expression. WAPL KO provides a new latency IIa B-cell model and lays the foundation for future studies of how WAPL remodels enhancer/promoter communication for EBV three-dimensional genome regulation, an area that is of interest to investigate for double stranded DNA viruses more broadly.

## Materials and methods

### RNA-seq

RNA poly-A enrichment was performed prior to library preparation and NGS. Reads were mapped to the GRCh37 human and Akata EBV genomes. Transcripts were quantified with Salmon [58]. A log_2_FC of > 0.6 and adjusted p-value < 0.05 were significant. DEGs were determined by DESeq2 [59].

### Hi-C

Hi-C was performed as described [27]. Significantly changed associations (FDR < 0.05 and Z-score > 1 or < -1) were plotted with circlize [60].

### HiChIP

HiChIP was performed as described [51]. Read loops between EBV genomic bins (1.5kb) were quantified and normalized using loops per 10k total read pairs. Differences were evaluated by Wilcoxon Rank Sum. Differential loops (p-value < 0.1, difference > 3 normalized read pairs, mean read pairs ≥ 2 in at least one condition) were visualized by circlize [60].

## Supporting information

S1 Text Supplementary methods. (DOCX)

S1 Fig Burkitt lymphoma cells and LCLs are viable upon CRISPR-mediated WAPL KO. **(A, B)** Representative immunoblot analysis (upper) demonstrating that sgRNA expression in Cas9+ cells leads to successful WAPL knockout in (A) MUTU I Burkitt lymphoma cells and (B) GM12878 LCLs, and CTG assay (lower) indicating that this knockout does not impede cell viability for either cell type. CTG plots show mean relative live cell number ± SD from 3 biological replicates. **(C, D)** Densitometry analysis of WAPL protein levels in (C) Cas9+ MUTU I and (D) Cas9+ GM12878 upon expression of control or WAPL sgRNAs. Densitometry values were normalized to GAPDH loading control values. Mean ± SD values from at least n = 3 biological replicates are shown. ** *P* ≤ 0.01, as calculated by a two-tailed Welch’s t-test. **(E, F)** Representative immunofluorescence images of anti-RAD21 (green) vs. nuclear DAPI (blue) staining in (E) Cas9+ MUTU I and (F) Cas9+ GM12878 cells expressing control or WAPL sgRNAs. Images are representative of 3 biological replicates. Scale bar is 5 μm. **(G)** Quantification of the number of copies of EBV genomes present in Cas9+ MUTU I cells expressing control or WAPL sgRNAs. Mean ± SD from at least 2 biological replicates. ns = not significant, as calculated by a two-tailed Student’s t-test. **(H)** Immunoblot analysis of WAPL and EBNA2 levels in Cas9+ MUTU I and GM12878 cells expressing control or WAPL sgRNAs. Immunoblots are representative of n = 3 biological replicates and densitometry values are shown. GAPDH-normalized WAPL levels in control lanes were normalized to 1, as were GAPDH-normalized EBNA2 levels in GM12878 control samples. ND = not detected. **(I)** Densitometry analysis of LMP1, LMP2A, EBNA1, and EBNA2 protein levels in Cas9+ MUTU I (upper) and Cas9+ GM12878 (lower) cells. Densitometry values were normalized to GAPDH loading control values. Mean ± SD values from at least n = 3 biological replicates are shown. * *P* ≤ 0.05, ns = not significant, as calculated by a two-tailed Welch’s t-test.
(TIF)

S2 Fig WAPL levels are higher in MUTU I (latency I) than in GM12878 (latency III). **(A)** Mean *WAPL* mRNA abundance ± SD from n = 3 biological RNA-seq replicates. **** *P* ≤ 0.001 as calculated by DESeq2 (see RNA-seq analysis methods). **(B)** Mean WAPL protein abundance ± SD from the n = 3 biological replicates shown in S1H Fig. Immunoblot WAPL levels were quantified by densitometry and normalized to GAPDH loading control levels. *P* value shown calculated by a two-tailed Student’s t-test. **(C-E)** Mean (C) *GAPDH* and *TUBA1B* (genes that are expressed equally in latency I and III), (D) *FAS* and *ICAM1* (genes that are expressed more highly in latency III than I), and (E) *BCL6* and *MME* (genes that are expressed more highly in latency I than III) mRNA abundance ± SD from n = 3 biological RNA-seq replicates. **** *P* ≤ 0.001, ns = not significant as calculated by DESeq2 (see RNA-seq analysis methods).
(TIF)

S3 Fig Burkitt cells and LCLs have similar changes in human gene expression upon CRISPR-mediated WAPL knockout. **(A)** Volcano plot of RNA-seq analysis visualizing -log_10_(adj. p-value) vs. log_2_(fold change of human mRNA abundance) from Cas9+ MUTU I Burkitt lymphoma cells expressing WAPL vs. control sgRNAs, from n = 3 independent biological replicates. **(B)** Significantly altered (p < 0.05) GO Biological Processes upon WAPL vs. control sgRNA expression in Cas9+ MUTU I cells. **(C)** Volcano plot of RNA-seq analysis visualizing -log_10_(adj. p-value) vs. log_2_(fold change of human mRNA abundance) from Cas9+ GM12878 LCLs expressing WAPL vs. control sgRNAs, from n = 3 independent biological replicates. **(D)** Significantly altered (p < 0.05) GO Biological Processes upon WAPL vs. control sgRNA expression in Cas9+ GM12878 LCLs.
(TIF)

S4 Fig WAPL loss leads to increased LMP1 and LMP2A levels. **(A)** Immunoblot analysis of LMP1 and LMP2A in Cas9+ P3HR-1 Burkitt lymphoma cells expressing WAPL or control sgRNAs. Immunoblot is representative of 3 biological replicates with densitometry values normalized to the loading control GAPDH shown. ND indicates not detected. **(B)** Representative immunofluorescence images from n = 3 biological replicates of anti-LMP1 (green) vs. nuclear DAPI (blue) staining of Cas9+ P3HR-1 cells that expressed control or WAPL sgRNAs, as indicated. Shown at right are zoomed images of a representative cell (indicated by the white box). **(C)** Mean ± SD percentage of LMP1+ cells per field of view, from n = 3 fields of view from each of three biological replicates. *P*-values shown as calculated by one-way ANOVA. **(D)** Representative immunofluorescence images from n = 3 biological replicates of anti-LMP2A (green) vs. nuclear DAPI (blue) staining of Cas9+ P3HR-1 cells that expressed control or WAPL sgRNAs, as indicated. Shown at right are zoomed images of a representative cell (indicated by the white box). **(E)** Mean ± SD percentage of LMP2A+ cells per field of view, from n = 3 fields of view from each of three biological replicates. *P*-values shown as calculated by one-way ANOVA.
(TIF)

S5 Fig Loss of WAPL alters looping and enhancer-promoter looping across the EBV genome. **(A)** H3K27ac HiChIP maps of all loops on the EBV genome that are enriched (red) or depleted (blue) in Cas9+ MUTU I cells expressing WAPL vs. control sgRNAs for each of the 3 biological replicates. **(B)** H3K27ac HiChIP maps of loops from the LMP promoter to other sites on the EBV genome that are enriched (red) or depleted (blue) in Cas9+ MUTU I cells expressing WAPL vs. control sgRNAs for each of the 3 biological replicates.
(TIF)

S6 Fig Effects of WAPL depletion on LMP1 and LMP2A promoter histone marks. **(A)** ChIP-qPCR analysis of CTCF and SMC1 cohesin abundances at the LMP promoter in Cas9+ MUTU I expressing control or WAPL sgRNAs. Shown are mean percentage of input ChIP-qPCR values ± SD from n = 3 biological replicates. * *P* ≤ 0.05, ** *P* ≤ 0.01, as calculated by a one-way ANOVA. **(B, C)** ChIP-qPCR analysis of H2AK119ub, H3K27ac, and H3K4me3 abundances at the (B) LMP1 promoter and (C) LMP2A promoter in Cas9+ MUTU I expressing control or WAPL sgRNAs. Shown in B-C are the mean fold change of ChIP-qPCR values relative to input values ± SD from n = 3 biological replicates. Values from sgControl expressing cells were normalized to 1. * *P* ≤ 0.05, ns = not significant, as calculated by a two-tailed Welch’s t-test.
(TIF)

S1 Table RNA-seq data. (XLSX)

S2 Table Hi-C data. (XLSX)

S3 Table HiChIP data. (XLSX)

## References

[1] Gewurz BE, Longnecker R, Cohen JI. Epstein-Barr Virus. 7th ed. In: Knipe D, Howley P, editors. Fields Virology. 7th ed. Wolters Kluwer; 2021. pp. 324–389.

[2] Farrell PJ. Epstein-Barr Virus and Cancer. Annu Rev Pathol. 2019;14: 29–53. doi: 10.1146/annurev-pathmechdis-012418-013023 30125149

[3] Young LS, Yap LF, Murray PG. Epstein-Barr virus: More than 50 years old and still providing surprises. Nat Rev Cancer. 2016;16: 789–802. doi: 10.1038/nrc.2016.92 27687982

[4] Damania B, Kenney SC, Raab-Traub N. Epstein-Barr virus: Biology and clinical disease. Cell. 2022;185: 3652–3670. doi: 10.1016/j.cell.2022.08.026 36113467 PMC9529843

[5] Yu H, Robertson ES. Epstein–Barr Virus History and Pathogenesis. Viruses. 2023;15: 714. doi: 10.3390/v15030714 36992423 PMC10056551

[6] Buschle A, Hammerschmidt W. Epigenetic lifestyle of Epstein-Barr virus. Semin Immunopathol. 2020;42: 131–142. doi: 10.1007/s00281-020-00792-2 32232535 PMC7174264

[7] Price AM, Luftig MA. To Be or Not IIb: A Multi-Step Process for Epstein-Barr Virus Latency Establishment and Consequences for B Cell Tumorigenesis. PLoS Pathog. 2015;11: 1–7. doi: 10.1371/journal.ppat.1004656 25790223 PMC4366242

[8] Murata T, Sugimoto A, Inagaki T, Yanagi Y, Watanabe T, Sato Y, et al. Molecular basis of Epstein–Barr virus latency establishment and lytic reactivation. Viruses. 2021;13: 1–20. doi: 10.3390/v13122344 34960613 PMC8706188

[9] Kieser A, Sterz KR. The latent membrane protein 1 (LMP1). Curr Top Microbiol Immunol. 2015;391: 119–149. doi: 10.1007/978-3-319-22834-1_4 26428373

[10] Wang LWL, Jiang S, Gewurz BE. Epstein-Barr Virus LMP1-Mediated Oncogenicity. J Virol. 2017;91: e01718–16. doi: 10.1128/JVI.01718-16 28835489 PMC5640852

[11] Fish K, Comoglio F, Shaffer AL, Ji Y, Pan KT, Scheich S, et al. Rewiring of B cell receptor signaling by Epstein-Barr virus LMP2A. Proc Natl Acad Sci U S A. 2020;117: 26318–26327. doi: 10.1073/pnas.2007946117 33020271 PMC7584892

[12] Liao Y, Yan J, Beri NR, Roth LG, Cesarman E, Gewurz BE. Germinal center cytokines driven epigenetic control of Epstein-Barr virus latency gene expression. PLoS Pathog. 2024;20: e1011939. doi: 10.1371/journal.ppat.1011939 38683861 PMC11081508

[13] Konforte D, Simard N, Paige CJ. Interleukin-21 regulates expression of key Epstein-Barr virus oncoproteins, EBNA2 and LMP1, in infected human B cells. Virology. 2008;374: 100–113. doi: 10.1016/j.virol.2007.12.027 18222514

[14] Kis LL, Takahara M, Nagy N, Klein G, Klein E. Cytokine mediated induction of the major Epstein-Barr virus (EBV)-encoded transforming protein, LMP-1. Immunol Lett. 2006;104: 83–88. doi: 10.1016/j.imlet.2005.11.003 16386314

[15] Li X, Bhaduri-McIntosh S. A central role for STAT3 in gammaherpesvirus-life cycle and -diseases. Front Microbiol. 2016;7: 1–10. doi: 10.3389/fmicb.2016.01052 27458446 PMC4937026

[16] Weniger MA, Küppers R. Molecular biology of Hodgkin lymphoma. Leukemia. 2021;35: 968–981. doi: 10.1038/s41375-021-01204-6 33686198 PMC8024192

[17] Yang J, Liu Z, Zeng B, Hu G, Gan R. Epstein–Barr virus-associated gastric cancer: A distinct subtype. Cancer Lett. 2020;495: 191–199. doi: 10.1016/j.canlet.2020.09.019 32979463

[18] Holdorf MM, Cooper SB, Yamamoto KR, Miranda JJL. Occupancy of chromatin organizers in the Epstein-Barr virus genome. Virology. 2011;415: 1–5. doi: 10.1016/j.virol.2011.04.004 21550623 PMC3808970

[19] Guo R, Gewurz BE. Epigenetic control of the Epstein-Barr lifecycle. Curr Opin Virol. 2022;52: 78–88. doi: 10.1016/j.coviro.2021.11.013 34891084 PMC9112224

[20] Lieberman P. Chromatin Structure of Epstein-Barr Virus Latent Episomes. Curr Top Microbiol Immunol. 2015;390: 71–102. doi: 10.1007/978-3-319-22822-8_5 26424644

[21] Caruso LB, Maestri D, Tempera I. Three-Dimensional Chromatin Structure of the EBV Genome: A Crucial Factor in Viral Infection. Viruses. 2023;15: 1–14. doi: 10.3390/v15051088 37243174 PMC10222312

[22] Haering CH, Farcas AM, Arumugam P, Metson J, Nasmyth K. The cohesin ring concatenates sister DNA molecules. Nature. 2008;454: 297–301. doi: 10.1038/nature07098 18596691

[23] Tempera I, Klichinsky M, Lieberman PM. EBV latency types adopt alternative chromatin conformations. PLoS Pathog. 2011;7: e1002180. doi: 10.1371/journal.ppat.1002180 21829357 PMC3145795

[24] Guo R, Jiang C, Zhang Y, Govande A, Trudeau SJ, Chen F, et al. MYC Controls the Epstein-Barr Virus Lytic Switch. Mol Cell. 2020;78: 653–669.e8. doi: 10.1016/j.molcel.2020.03.025 32315601 PMC7245572

[25] Ding W, Wang C, Narita Y, Wang H, Leong MML, Huang A, et al. The Epstein-Barr Virus Enhancer Interaction Landscapes in Virus-Associated Cancer Cell Lines. J Virol. 2022;96: e0073922. doi: 10.1128/jvi.00739-22 36094314 PMC9517713

[26] Tempera I, Lieberman PM. Chromatin organization of gammaherpesvirus latent genomes. Biochim Biophys Acta—Gene Regul Mech. 2010;1799: 236–245. doi: 10.1016/j.bbagrm.2009.10.004 19853673 PMC2839031

[27] Morgan SM, Tanizawa H, Caruso LB, Hulse M, Kossenkov A, Madzo J, et al. The three-dimensional structure of Epstein-Barr virus genome varies by latency type and is regulated by PARP1 enzymatic activity. Nat Commun. 2022;13: 187. doi: 10.1038/s41467-021-27894-1 35039491 PMC8764100

[28] Hughes DJ, Marendy EM, Dickerson CA, Yetming KD, Sample CE, Sample JT. Contributions of CTCF and DNA Methyltransferases DNMT1 and DNMT3B to Epstein-Barr Virus Restricted Latency. J Virol. 2012;86: 1034–1045. doi: 10.1128/JVI.05923-11 22072770 PMC3255836

[29] Lee SH, Kim KD, Cho M, Huh S, An SH, Seo D, et al. Characterization of a new CCCTC-binding factor binding site as a dual regulator of Epstein-Barr virus latent infection. PLoS Pathog. 2023;19: 1–32. doi: 10.1371/journal.ppat.1011078 36696451 PMC9876287

[30] Chen H-S, Martin KA, Lu F, Lupey LN, Mueller JM, Lieberman PM, et al. Epigenetic Deregulation of the LMP1/LMP2 Locus of Epstein-Barr Virus by Mutation of a Single CTCF-Cohesin Binding Site. J Virol. 2014;88: 1703–1713. doi: 10.1128/JVI.02209-13 24257606 PMC3911611

[31] Salamon D, Banati F, Koroknai A, Ravasz M, Szenthe K, Bathori Z, et al. Binding of CCCTC-binding factor in vivo to the region located between Rep* and the C promoter of Epstein-Barr virus is unaffected by CpG methylation and does not correlate with Cp activity. J Gen Virol. 2009;90: 1183–1189. doi: 10.1099/vir.0.007344-0 19264589

[32] Arvey A, Tempera I, Tsai K, Chen HS, Tikhmyanova N, Klichinsky M, et al. An atlas of the Epstein-Barr virus transcriptome and epigenome reveals host-virus regulatory interactions. Cell Host Microbe. 2012;12: 233–245. doi: 10.1016/j.chom.2012.06.008 22901543 PMC3424516

[33] Haarhuis JHI, van der Weide RH, Blomen VA, Yáñez-Cuna JO, Amendola M, van Ruiten MS, et al. The Cohesin Release Factor WAPL Restricts Chromatin Loop Extension. Cell. 2017;169: 693–707.e14. doi: 10.1016/j.cell.2017.04.013 28475897 PMC5422210

[34] Tedeschi A, Wutz G, Huet S, Jaritz M, Wuensche A, Schirghuber E, et al. Wapl is an essential regulator of chromatin structure and chromosome segregation. Nature. 2013;501: 564–568. doi: 10.1038/nature12471 23975099 PMC6080692

[35] Beckouët F, Srinivasan M, Roig MB, Chan KL, Scheinost JC, Batty P, et al. Releasing Activity Disengages Cohesin’s Smc3/Scc1 Interface in a Process Blocked by Acetylation. Mol Cell. 2016;61: 563–574. doi: 10.1016/j.molcel.2016.01.026 26895425 PMC4769318

[36] Murayama Y, Uhlmann F. DNA Entry into and Exit out of the Cohesin Ring by an Interlocking Gate Mechanism. Cell. 2015;163: 1628–1640. doi: 10.1016/j.cell.2015.11.030 26687354 PMC4701713

[37] Kwiatkowski BA, Ragoczy T, Ehly J, Schubach WH. Identification and cloning of a novel chromatin-associated protein partner of Epstein-Barr nuclear protein 2. Exp Cell Res. 2004;300: 223–233. doi: 10.1016/j.yexcr.2004.06.028 15383329

[38] Mitra B, Beri NR, Guo R, Burton EM, Murray-Nerger LA, Gewurz BE. Characterization of target gene regulation by the two Epstein-Barr virus oncogene LMP1 domains essential for B-cell transformation. MBio. 2023;14: e0233823. doi: 10.1128/mbio.02338-23 38009935 PMC10746160

[39] Longnecker R, Kieff E. A second Epstein-Barr virus membrane protein (LMP2) is expressed in latent infection and colocalizes with LMP1. J Virol. 1990;64: 2319–2326. doi: 10.1128/JVI.64.5.2319-2326.1990 2157888 PMC249393

[40] Longnecker R, Druker B, Roberts TM, Kieff E. An Epstein-Barr virus protein associated with cell growth transformation interacts with a tyrosine kinase. J Virol. 1991;65: 3681–3692. doi: 10.1128/JVI.65.7.3681-3692.1991 1710288 PMC241385

[41] Lam N, Sugden B. LMP1, a viral relative of the TNF receptor family, signals principally from intracellular compartments. EMBO J. 2003;22: 3027–3038. doi: 10.1093/emboj/cdg284 12805217 PMC162136

[42] Meckes DG, Menaker NF, Raab-Traub N. Epstein-Barr Virus LMP1 Modulates Lipid Raft Microdomains and the Vimentin Cytoskeleton for Signal Transduction and Transformation. J Virol. 2013;87: 1301–1311. doi: 10.1128/JVI.02519-12 23152522 PMC3554135

[43] Wang LW, Wang Z, Ersing I, Nobre L, Guo R, Jiang S, et al. Epstein-Barr virus subverts mevalonate and fatty acid pathways to promote infected B-cell proliferation and survival. PLoS Pathog. 2019;15: 1–35. doi: 10.1371/journal.ppat.1008030 31518366 PMC6760809

[44] Liebowitz D, Wang D, Kieff E. Orientation and patching of the latent infection membrane protein encoded by Epstein-Barr virus. J Virol. 1986;58: 233–237. doi: 10.1128/JVI.58.1.233-237.1986 3005654 PMC252901

[45] King W, Dambaugh T, Heller M, Dowling J, Kieff E. Epstein-Barr virus DNA XII. A variable region of the Epstein-Barr virus genome is included in the P3HR-1 deletion. J Virol. 1982;43: 979–986. doi: 10.1128/JVI.43.3.979-986.1982 6292475 PMC256208

[46] Bornkamm GW, Hudewentz J, Freese UK, Zimber U. Deletion of the Nontransforming Epstein-Barr Virus Strain P3HR-1 Causes Fusion of the Large Internal Repeat to the DS L Region. J Virol. 1982;43: 952–968. doi: 10.1128/JVI.43.3.952-968.1982 6292473 PMC256206

[47] Rabson M, Gradoville L, Heston L, Miller G. Non-immortalizing P3J-HR-1 Epstein-Barr virus: a deletion mutant of its transforming parent, Jijoye. J Virol. 1982;44: 834–844. doi: 10.1128/JVI.44.3.834-844.1982 6294333 PMC256340

[48] Rowe D, Heston L, Metlay J, Miller G. Identification and expression of a nuclear antigen from the genomic region of the Jijoye strain of Epstein-Barr virus that is missing in its nonimmortalizing deletion mutant, P3HR-1. Proc Natl Acad Sci U S A. 1985;82: 7429–7433. doi: 10.1073/pnas.82.21.7429 2997790 PMC391358

[49] Lajoie BR, Dekker J, Kaplan N. The Hitchhiker’s guide to Hi-C analysis: Practical guidelines. Methods. 2015;72: 65–75. doi: 10.1016/j.ymeth.2014.10.031 25448293 PMC4347522

[50] Rao SSP, Huntley MH, Durand NC, Stamenova EK, Bochkov ID, Robinson JT, et al. A 3D map of the human genome at kilobase resolution reveals principles of chromatin looping. Cell. 2014;159: 1665–1680. doi: 10.1016/j.cell.2014.11.021 25497547 PMC5635824

[51] Mumbach MR, Rubin AJ, Flynn RA, Dai C, Khavari PA, Greenleaf WJ, et al. HiChIP: Efficient and sensitive analysis of protein-directed genome architecture. Nat Methods. 2016;13: 919–922. doi: 10.1038/nmeth.3999 27643841 PMC5501173

[52] Creyghton MP, Cheng AW, Welstead GG, Kooistra T, Carey BW, Steine EJ, et al. Histone H3K27ac separates active from poised enhancers and predicts developmental state. Proc Natl Acad Sci U S A. 2010;107: 21931–21936. doi: 10.1073/pnas.1016071107 21106759 PMC3003124

[53] Jenuwein T, Allis CD. Translating the histone code. Science (80-). 2001;293: 1074–1080. doi: 10.1126/science.1063127 11498575

[54] Guo R, Zhang Y, Teng M, Jiang C, Schineller M, Zhao B, et al. DNA methylation enzymes and PRC1 restrict B-cell Epstein–Barr virus oncoprotein expression. Nat Microbiol. 2020;5: 1051–1063. doi: 10.1038/s41564-020-0724-y 32424339 PMC7462085

[55] Maestri D, Napoletani G, Kossenkov A, Preston-Alp S, Caruso LB, Tempera I. The three-dimensional structure of the EBV genome plays a crucial role in regulating viral gene expression in EBVaGC. Nucleic Acids Res. 2023;51: 12092–12110. doi: 10.1093/nar/gkad936 37889078 PMC10711448

[56] Lu F, Wiedmer A, Martin KA, Wickramasinghe PJMS, Kossenkov A V., Lieberman PM. Coordinate Regulation of TET2 and EBNA2 Controls the DNA Methylation State of Latent Epstein-Barr Virus. J Virol. 2017;91: e00804–17. doi: 10.1128/JVI.00804-17 28794029 PMC5625499

[57] Wille C, Li Y, Rui L, Johannsen E, Kenney S. Restricted TET2 Expression in Germinal Center Type B Cells Promotes Stringent Epstein-Barr Virus Latency. J Virol. 2017;91: e01987–16. doi: 10.1128/JVI.01987-16 28003489 PMC5309966

[58] Patro R, Duggal G, Love MI, Irizarry RA, Kingsford C. Salmon provides fast and bias-aware quantification of transcript expression. Nat Methods. 2017;14: 417–419. doi: 10.1038/nmeth.4197 28263959 PMC5600148

[59] Love MI, Huber W, Anders S. Moderated estimation of fold change and dispersion for RNA-seq data with DESeq2. Genome Biol. 2014;15: 1–21. doi: 10.1186/s13059-014-0550-8 25516281 PMC4302049

[60] Gu Z, Gu L, Eils R, Schlesner M, Brors B. Circlize implements and enhances circular visualization in R. Bioinformatics. 2014;30: 2811–2812. doi: 10.1093/bioinformatics/btu393 24930139

