# Supplementary figures and images for: The DNA loop release factor WAPL suppresses Epstein-Barr virus latent membrane protein expression to maintain the highly restricted latency I program
Source: PLoS Pathog. 2024 Sep 6;20(9):e1012525. doi: 10.1371/journal.ppat.1012525 (PMC11410233; doi:10.1371/journal.ppat.1012525)

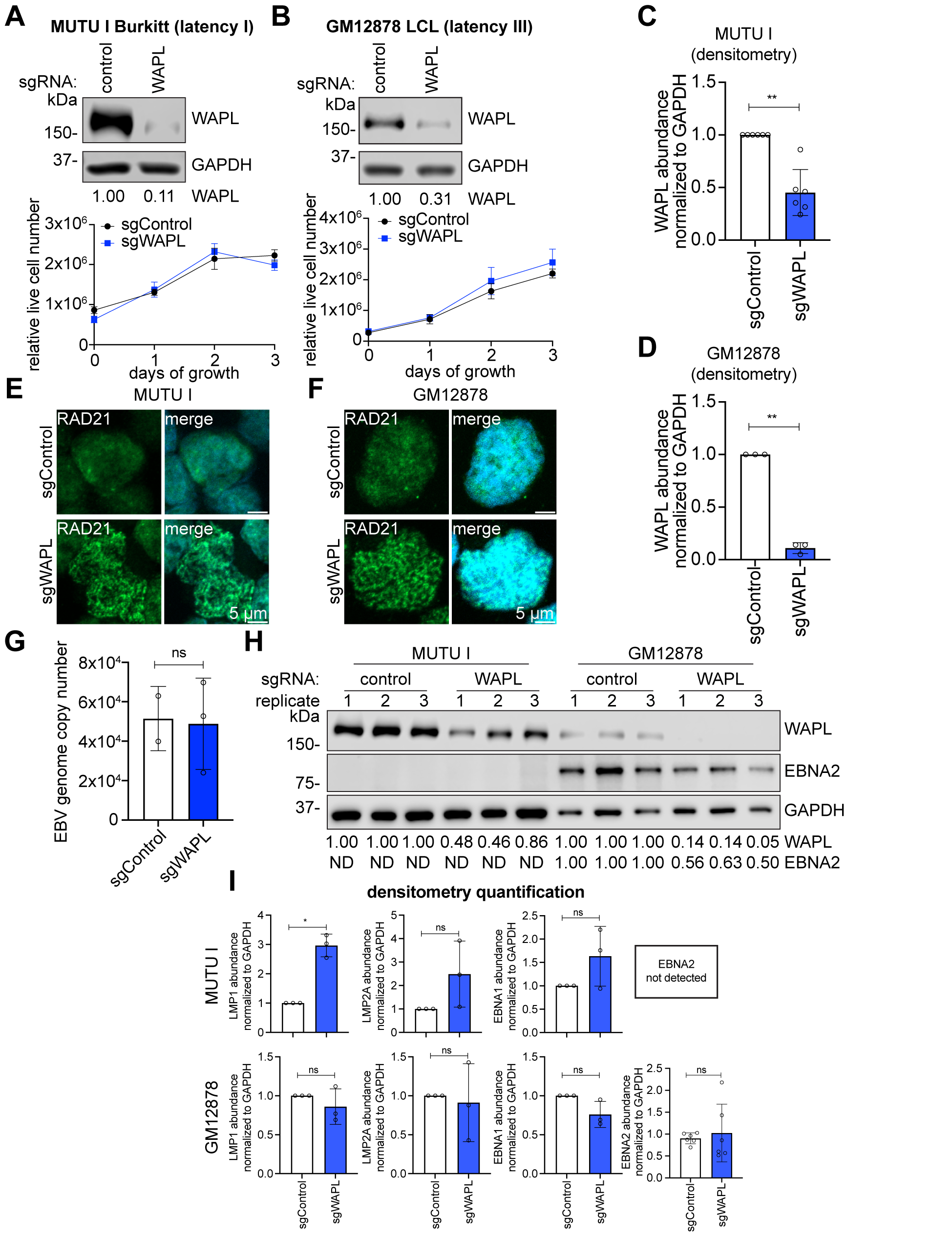

Supplement: S1 Fig — (A, B) Representative immunoblot analysis (upper) demonstrating that sgRNA expression in Cas9+ cells leads to successful WAPL knockout in (A) MUTU I Burkitt lymphoma cells and (B) GM12878 LCLs, and CTG assay (lower) indicating that this knockout does not impede cell viability for either cell type. CTG plots show mean relative live cell number ± SD from 3 biological replicates. (C, D) Densitometry analysis of WAPL protein levels in (C) Cas9+ MUTU I and (D) Cas9+ GM12878 upon expression of control or WAPL sgRNAs. Densitometry values were normalized to GAPDH loading control values. Mean ± SD values from at least n = 3 biological replicates are shown. ** P ≤ 0.01, as calculated by a two-tailed Welch’s t-test. (E, F) Representative immunofluorescence images of anti-RAD21 (green) vs. nuclear DAPI (blue) staining in (E) Cas9+ MUTU I and (F) Cas9+ GM12878 cells expressing control or WAPL sgRNAs. Images are representative of 3 biological replicates. Scale bar is 5 μm. (G) Quantification of the number of copies of EBV genomes present in Cas9+ MUTU I cells expressing control or WAPL sgRNAs. Mean ± SD from at least 2 biological replicates. ns = not significant, as calculated by a two-tailed Student’s t-test. (H) Immunoblot analysis of WAPL and EBNA2 levels in Cas9+ MUTU I and GM12878 cells expressing control or WAPL sgRNAs. Immunoblots are representative of n = 3 biological replicates and densitometry values are shown. GAPDH-normalized WAPL levels in control lanes were normalized to 1, as were GAPDH-normalized EBNA2 levels in GM12878 control samples. ND = not detected. (I) Densitometry analysis of LMP1, LMP2A, EBNA1, and EBNA2 protein levels in Cas9+ MUTU I (upper) and Cas9+ GM12878 (lower) cells. Densitometry values were normalized to GAPDH loading control values. Mean ± SD values from at least n = 3 biological replicates are shown. * P ≤ 0.05, ns = not significant, as calculated by a two-tailed Welch’s t-test. (TIF) [file ppat.1012525.s002.tif]

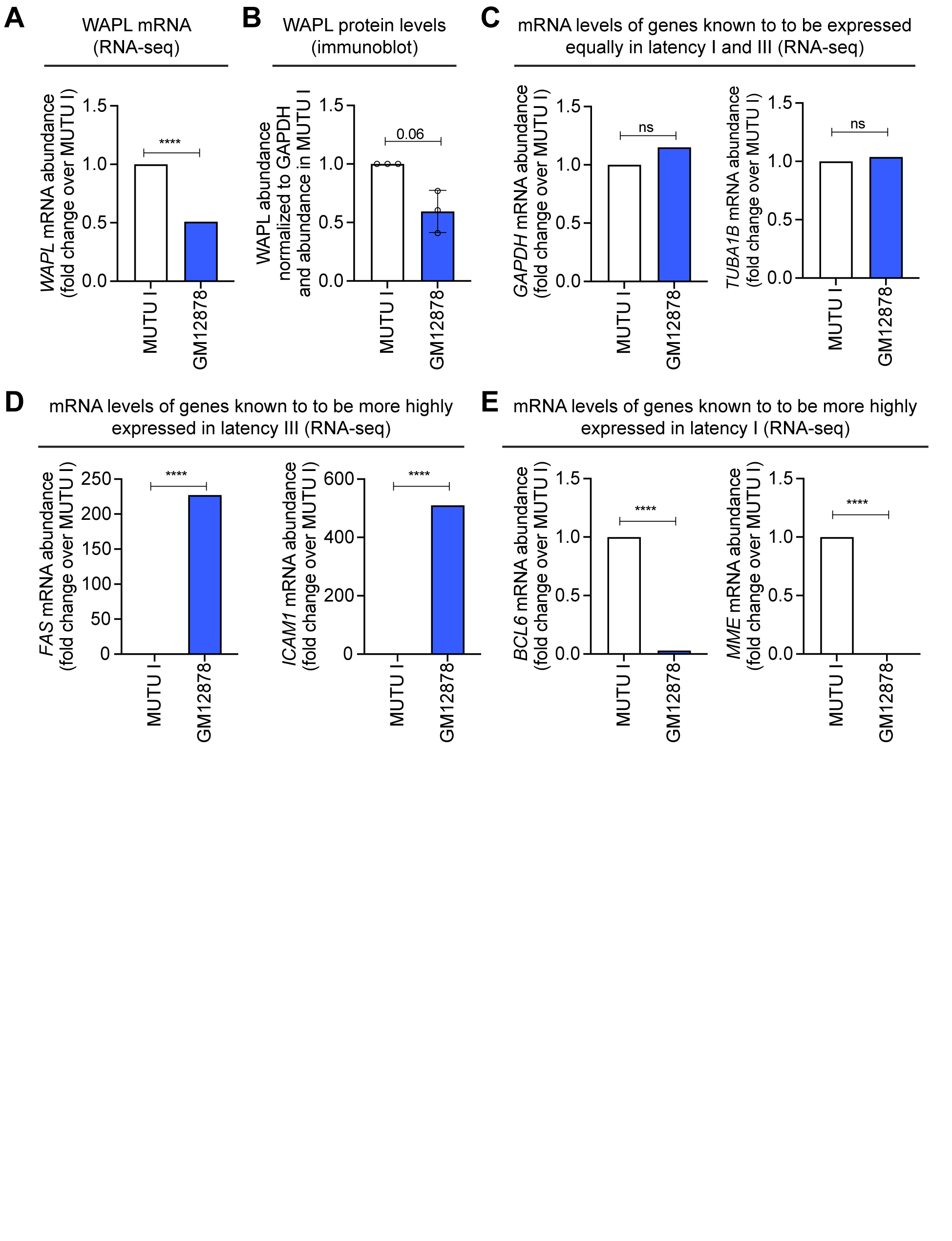

Supplement: S2 Fig — (A) Mean WAPL mRNA abundance ± SD from n = 3 biological RNA-seq replicates. **** P ≤ 0.001 as calculated by DESeq2 (see RNA-seq analysis methods). (B) Mean WAPL protein abundance ± SD from the n = 3 biological replicates shown in S1H Fig. Immunoblot WAPL levels were quantified by densitometry and normalized to GAPDH loading control levels. P value shown calculated by a two-tailed Student’s t-test. (C-E) Mean (C) GAPDH and TUBA1B (genes that are expressed equally in latency I and III), (D) FAS and ICAM1 (genes that are expressed more highly in latency III than I), and (E) BCL6 and MME (genes that are expressed more highly in latency I than III) mRNA abundance ± SD from n = 3 biological RNA-seq replicates. **** P ≤ 0.001, ns = not significant as calculated by DESeq2 (see RNA-seq analysis methods). (TIF) [file ppat.1012525.s003.tif]

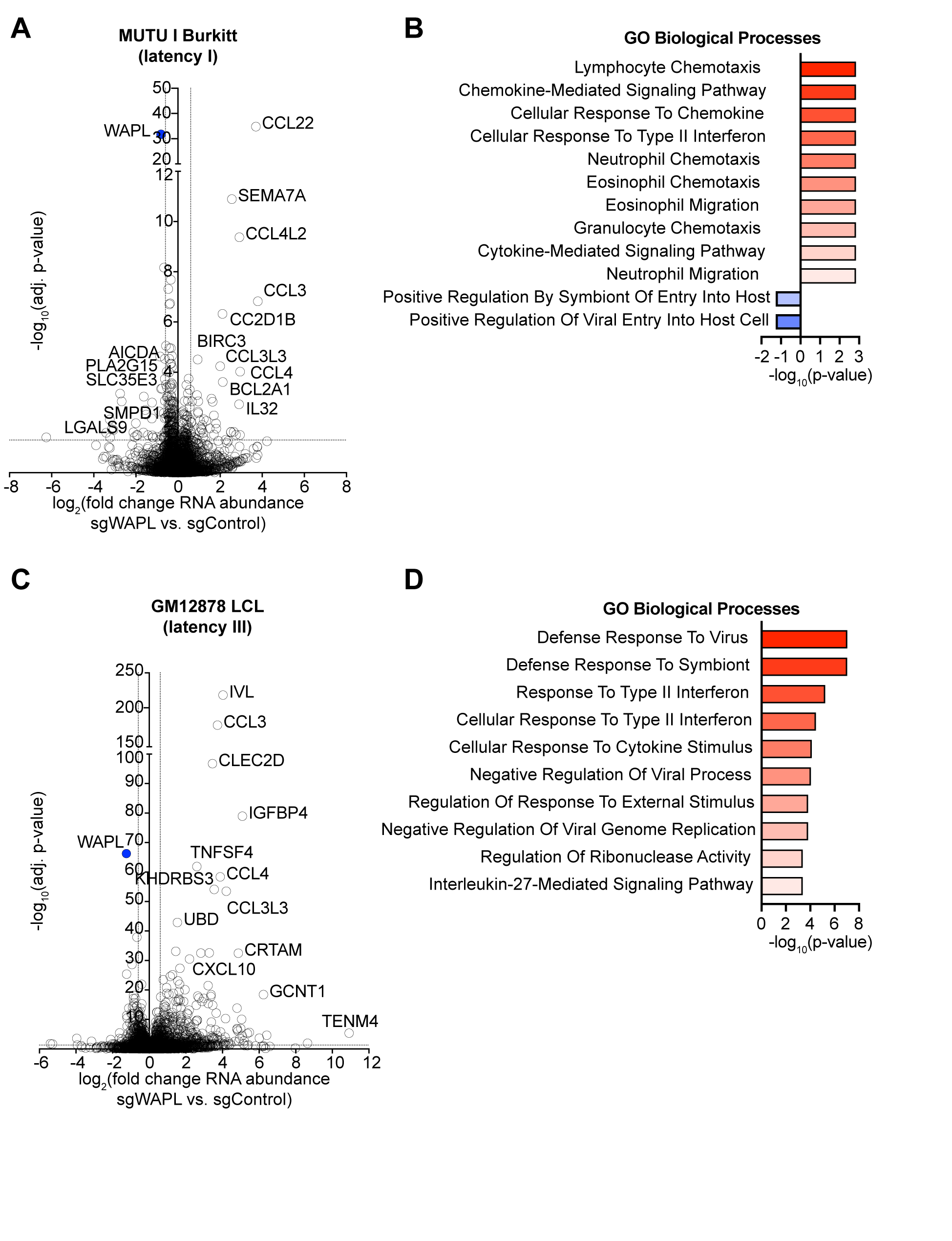

Supplement: S3 Fig — (A) Volcano plot of RNA-seq analysis visualizing -log10(adj. p-value) vs. log2(fold change of human mRNA abundance) from Cas9+ MUTU I Burkitt lymphoma cells expressing WAPL vs. control sgRNAs, from n = 3 independent biological replicates. (B) Significantly altered (p < 0.05) GO Biological Processes upon WAPL vs. control sgRNA expression in Cas9+ MUTU I cells. (C) Volcano plot of RNA-seq analysis visualizing -log10(adj. p-value) vs. log2(fold change of human mRNA abundance) from Cas9+ GM12878 LCLs expressing WAPL vs. control sgRNAs, from n = 3 independent biological replicates. (D) Significantly altered (p < 0.05) GO Biological Processes upon WAPL vs. control sgRNA expression in Cas9+ GM12878 LCLs. (TIF) [file ppat.1012525.s004.tif]

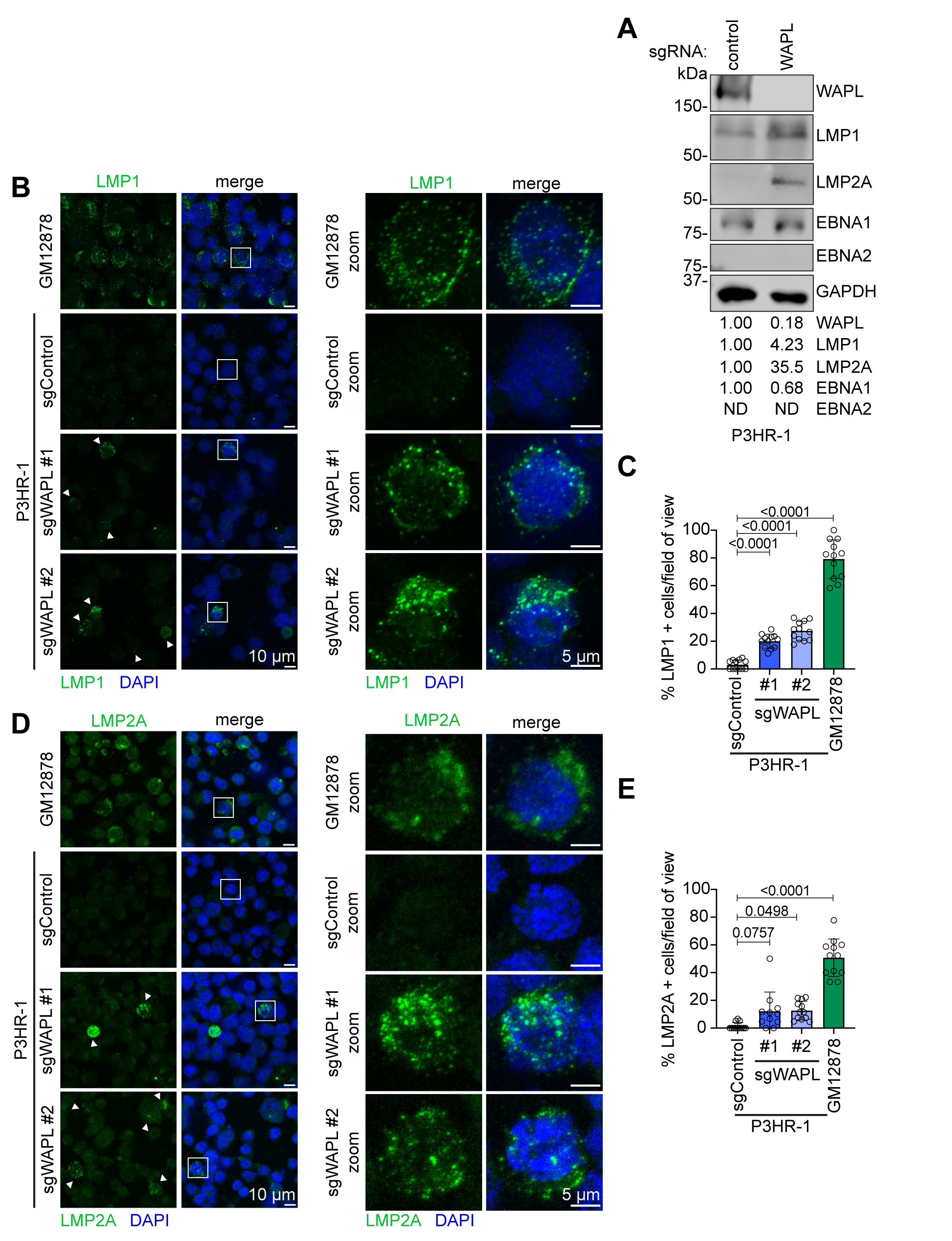

Supplement: S4 Fig — (A) Immunoblot analysis of LMP1 and LMP2A in Cas9+ P3HR-1 Burkitt lymphoma cells expressing WAPL or control sgRNAs. Immunoblot is representative of 3 biological replicates with densitometry values normalized to the loading control GAPDH shown. ND indicates not detected. (B) Representative immunofluorescence images from n = 3 biological replicates of anti-LMP1 (green) vs. nuclear DAPI (blue) staining of Cas9+ P3HR-1 cells that expressed control or WAPL sgRNAs, as indicated. Shown at right are zoomed images of a representative cell (indicated by the white box). (C) Mean ± SD percentage of LMP1+ cells per field of view, from n = 3 fields of view from each of three biological replicates. P-values shown as calculated by one-way ANOVA. (D) Representative immunofluorescence images from n = 3 biological replicates of anti-LMP2A (green) vs. nuclear DAPI (blue) staining of Cas9+ P3HR-1 cells that expressed control or WAPL sgRNAs, as indicated. Shown at right are zoomed images of a representative cell (indicated by the white box). (E) Mean ± SD percentage of LMP2A+ cells per field of view, from n = 3 fields of view from each of three biological replicates. P-values shown as calculated by one-way ANOVA. (TIF) [file ppat.1012525.s005.tif]

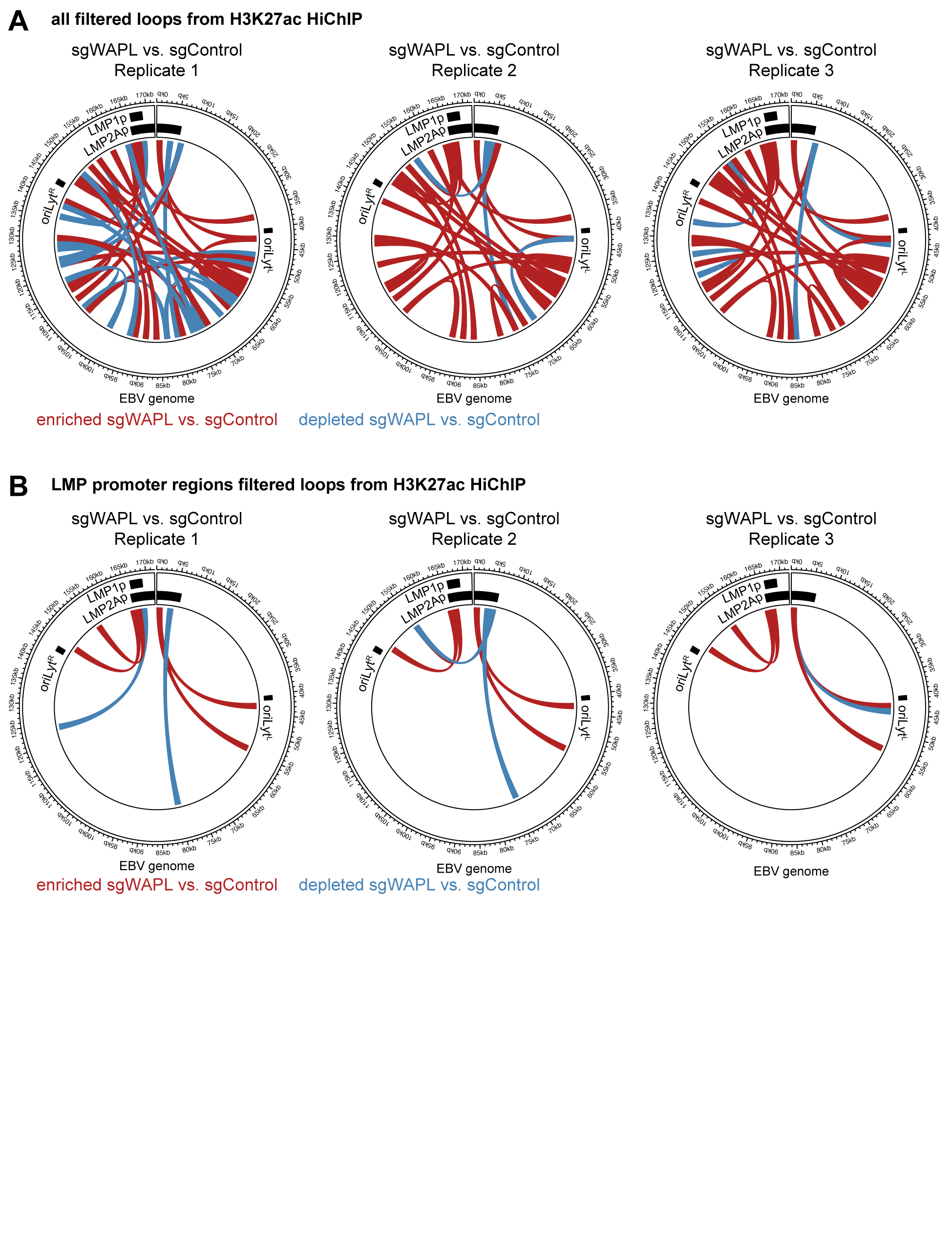

Supplement: S5 Fig — (A) H3K27ac HiChIP maps of all loops on the EBV genome that are enriched (red) or depleted (blue) in Cas9+ MUTU I cells expressing WAPL vs. control sgRNAs for each of the 3 biological replicates. (B) H3K27ac HiChIP maps of loops from the LMP promoter to other sites on the EBV genome that are enriched (red) or depleted (blue) in Cas9+ MUTU I cells expressing WAPL vs. control sgRNAs for each of the 3 biological replicates. (TIF) [file ppat.1012525.s006.tif]

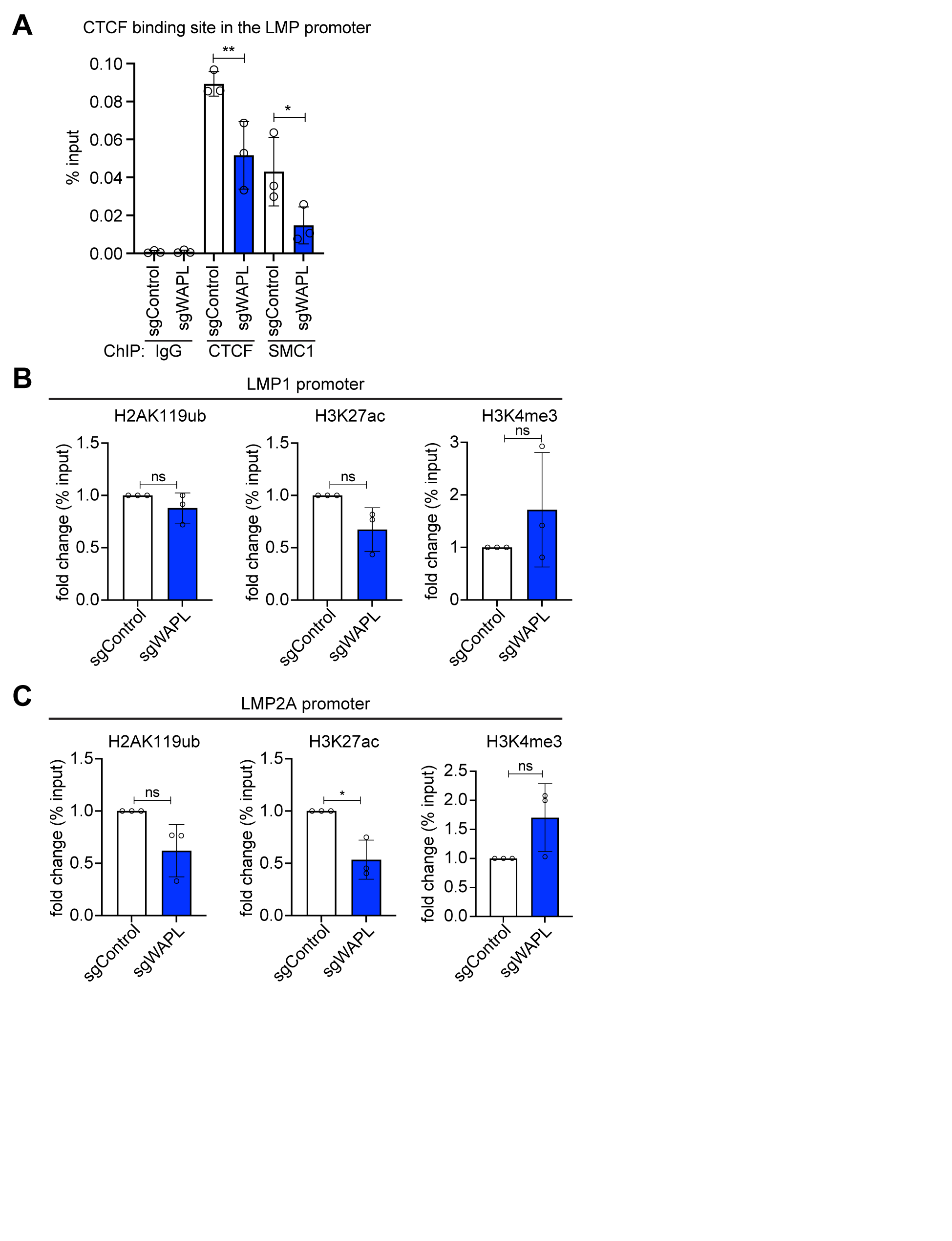

Supplement: S6 Fig — (A) ChIP-qPCR analysis of CTCF and SMC1 cohesin abundances at the LMP promoter in Cas9+ MUTU I expressing control or WAPL sgRNAs. Shown are mean percentage of input ChIP-qPCR values ± SD from n = 3 biological replicates. * P ≤ 0.05, ** P ≤ 0.01, as calculated by a one-way ANOVA. (B, C) ChIP-qPCR analysis of H2AK119ub, H3K27ac, and H3K4me3 abundances at the (B) LMP1 promoter and (C) LMP2A promoter in Cas9+ MUTU I expressing control or WAPL sgRNAs. Shown in B-C are the mean fold change of ChIP-qPCR values relative to input values ± SD from n = 3 biological replicates. Values from sgControl expressing cells were normalized to 1. * P ≤ 0.05, ns = not significant, as calculated by a two-tailed Welch’s t-test. (TIF) [file ppat.1012525.s007.tif]
